# Reciprocal links between anxiety sensitivity and obsessive–compulsive symptoms in youth: a longitudinal twin study

**DOI:** 10.1111/jcpp.13183

**Published:** 2020-01-16

**Authors:** G. Krebs, L.J. Hannigan, A.M. Gregory, F.V. Rijsdijk, T.C. Eley

**Affiliations:** ^1^ MRC Social, Genetic and Developmental Psychiatry Centre Institute of Psychiatry, Psychology & Neuroscience King's College London London UK; ^2^ National and Specialist OCD, BDD and Related Disorders Clinic for Young People South London and Maudsley NHS Foundation Trust London UK; ^3^ Nic Waals Institute Lovisenberg Diaconal Hospital Oslo Norway; ^4^ Department of Psychology Goldsmiths, University of London London UK

**Keywords:** Obsessive–compulsive disorder, anxiety sensitivity, adolescence, aetiology, genetics

## Abstract

**Background:**

Anxiety sensitivity, the tendency to fear the symptoms of anxiety, is a key risk factor for the development anxiety disorders. Although obsessive–compulsive disorder was previously classified as an anxiety disorder, the prospective relationship between anxiety sensitivity and obsessive–compulsive symptoms (OCS) has been largely overlooked. Furthermore, a lack of genetically informative studies means the aetiology of the link between anxiety sensitivity and OCS remains unclear.

**Methods:**

Adolescent twins and siblings (*N* = 1,579) from the G1219 study completed self‐report questionnaires two years apart assessing anxiety sensitivity, OCS, anxiety and depression. Linear regression models tested prospective associations between anxiety sensitivity and OCS, with and without adjustment for anxiety and depressive symptoms. A phenotypic cross‐lagged model assessed bidirectional influences between anxiety sensitivity and OCS over time, and a genetic version of this model examined the aetiology of these associations.

**Results:**

Anxiety sensitivity was prospectively associated with changes in OCS, even after controlling for comorbid anxiety and depressive symptoms. The longitudinal relationship between anxiety sensitivity and OCS was bidirectional, and these associations were predominantly accounted for by nonshared environmental influences.

**Conclusions:**

Our findings are consistent with the notion that anxiety sensitivity is a risk factor for OCS during adolescence, but also suggest that experiencing OCS confers risk for heightened anxiety sensitivity. The reciprocal links between OCS and anxiety sensitivity over time are likely to be largely mediated by nonshared environmental experiences, as opposed to common genes. Our findings raise the possibility that interventions aimed at ameliorating anxiety sensitivity could reduce risk for OCS, and vice versa.

## Introduction

Obsessive–compulsive disorder (OCD) is a debilitating condition affecting an estimated 2% of the population, but a much higher proportion experience subclinical levels of obsessions and compulsions (Ruscio, Stein, Chiu, & Kessler, 2010). These subclinical obsessive–compulsive symptoms (OCS) commonly emerge during youth and are associated with impairment in their own right (Fullana et al., 2009). Furthermore, OCS in childhood predict subsequent onset of OCD (Fullana et al., 2009). Thus, identifying mechanisms underpinning the development of OCS could have important implications for OCD prevention.

Anxiety sensitivity, the tendency to perceive symptoms of anxiety as being harmful, has been postulated as a cognitive risk factor for the development of anxiety disorders. Anxiety sensitivity predicts variance in anxiety symptoms independent of trait anxiety, demonstrating these are separate constructs (Olatunji & Wolitzky‐Taylor, 2009). Furthermore, anxiety sensitivity has been shown to positively predict the subsequent onset of a range of anxiety subtypes, over and above concurrent symptoms of anxiety (Schmidt, Zvolensky, & Maner, 2006; Waszczuk, Zavos, & Eley, 2013; Weems, Hayward, Killen, & Taylor, 2002), suggesting that it is a broad risk factor for anxiety disorders. While some studies have found the longitudinal relationship between anxiety sensitivity and anxiety symptoms to be unidirectional (Waszczuk et al., 2013), others have found evidence for bidirectionality (Schmidt, Lerew, & Joiner, 2000; Zavos, Rijsdijk, & Eley, 2012). In the bidirectional model, a negative perception of anxious arousal may render individuals vulnerable to developing anxiety symptoms, but experiencing anxiety symptoms may also lead to increased fear of anxious arousal (Zavos et al., 2012), resulting in a vicious cycle that perpetuates anxiety and leads to escalating symptoms.

Despite the fact that OCD was previously classified as an anxiety disorder (American Psychiatric Association, 2000; World Health Organization, 1992), little empirical attention has been given to the potential role of anxiety sensitivity in the development of OCD. From a theoretical perspective, it has been suggested that anxiety sensitivity may heighten fear conditioning and avoidance (Reiss, 1991), which are likely to be relevant mechanisms in the development of OCD (Abramowitz & Arch, 2014). Furthermore, individuals with high anxiety sensitivity may be predisposed to interpreting normal intrusive thoughts as threatening (Calamari, Rector, Woodard, Cohen, & Chik, 2008; Robinson & Freeston, 2014), which is a cardinal feature of OCD (Abramowitz & Arch, 2014). A number of cross‐sectional studies have indeed found that OCD patients display increased anxiety sensitivity relative to healthy controls (e.g. Wheaton, Deacon, McGrath, Berman, & Abramowitz, 2012). Similarly, anxiety sensitivity has been positively associated with continuous measures of OCS in clinical (e.g. Raines, Oglesby, Capron, & Schmidt, 2014) and nonclinical samples (Wheaton, Mahaffey, Timpano, Berman, & Abramowitz, 2012).

Despite preliminary evidence for a link between anxiety sensitivity and OCS, there are several notable limitations of previous studies. First, most have focused on adults, despite the fact that adolescence is known to be a crucial period for the development of OCS (Fullana et al., 2009) and therefore an ideal time for investigating aetiological mechanisms. Second, the prospective link between anxiety sensitivity and OCS has been rarely explored. In the only previous prospective study, anxiety sensitivity at age 11 was found to predict changes in OCS over a 12‐month period (Schmidt et al., 2010). Whether these findings extend to longer follow‐up periods and other stages of development remains unclear. Furthermore, research is needed to test whether the longitudinal association between anxiety sensitivity and OCS is unidirectional, or these two traits mutually influence each other over time (Zavos et al., 2012).

A further limitation of the existing literature is that no previous studies have examined the aetiology of the link between OCS and anxiety sensitivity – that is the extent to which it is underpinned by genetic and environmental factors. One possibility is that the association largely reflects genetic influences, with the same genes impacting on anxiety sensitivity and OCS. In this vein, previous twin studies have shown high genetic correlations between anxiety sensitivity and anxiety symptoms both concurrently (Zavos, Rijsdijk, Gregory, & Eley, 2010) and longitudinally (Waszczuk et al., 2013; Zavos et al., 2012). Alternatively, the relationship between anxiety sensitivity and OCS could be predominantly mediated by environmental factors. For example, individuals with elevated anxiety sensitivity could be vulnerable to developing OCS when exposed to stressful life events, in keeping with cognitive behavioural theories (Beck & Haigh, 2014).

In summary, the extent and aetiology of the longitudinal associations between anxiety sensitivity and OCS during adolescence remain unclear. The current study addressed three related aims. First, we sought to establish whether anxiety sensitivity prospectively predicted OCS during adolescence. Second, we aimed to determine whether anxiety sensitivity and OCS reciprocally influence each other over time (i.e. a bidirectional relationship). Third, we used genetic models to examine whether longitudinal associations between anxiety sensitivity and OCS are due to genetic and/or environmental influences.

## Method

### Participants

Participants were taken from the Genesis1219 project (G1219), a longitudinal study of twins and siblings born in the United Kingdom. Details of recruitment and selection have been described previously (McAdams et al., 2013). Ethical approval for the study was given by the Research Ethics Committee of the Institute of Psychiatry and South London and Maudsley NHS Trust, and informed consent was obtained from parents of adolescents under 16 years and from participants over 16. Zygosity of twins was determined using parental ratings of physical similarity across two time points (Price et al., 2000).

The current study included data from waves 2 and 3 of G1219, hereafter referred to as Time 1 and Time 2, respectively. Participants who completed the OCS measure at Time 2 were included in the current study, resulting in a sample of 1,579 twins and siblings (439 monozygotic (MZ) twins, 816 dizygotic (DZ) twins, 299 full siblings (FS) and 14 unknown), of whom 60% were female. The mean age was 14 years 11 months (*SD* = 1.64; range = 12–21) at Time 1 and 17 years (*SD* = 1.64; range = 14–23) at Time 2.

### Measures

#### Anxiety sensitivity

The self‐report Child Anxiety Sensitivity Index (CASI; Silverman, Fleisig, Rabian, & Peterson, 1991) comprises 18 items, which are scored on a 3‐point scale (i.e. total score of 18–54.) Items assess the extent to which children interpret anxiety symptoms as having negative physical, mental and social consequences (e.g. ‘When I notice that my heart is beating fast it scares me’). The scale has good construct validity, internal consistency and test–retest reliability (Silverman et al., 1991). Internal consistency in the current sample was .82 and .86 at Times 1 and 2, respectively.

#### Obsessive–compulsive and anxiety symptoms

The self‐report version of the Spence Children’s Anxiety Scale (SCAS) was used to measure obsessive–compulsive and anxiety symptoms (Spence, Barrett, & Turner, 2003). The scale contains 38 items scored on a 4‐point scale. The OCS subscale comprises six items assessing a range of compulsions (e.g. checking, repeating and mental rituals) and obsessions (e.g. fear of negative events and experiencing negative mental pictures), and yields a total score of 0–18. The OCS subscale has good internal consistency and adequate test–retest reliability, ranging across studies from .53 over 6 months (Spence, 1998) to .77 over 2 months (Essau, Anastassiou‐Hadjicharalambous, & Muñoz, 2011). The subscale also correlates well with the Children’s Yale‐Brown Obsessive‐Compulsive Scale (Whiteside, Gryczkowski, Biggs, Fagen, & Owusu, 2012). Internal consistency in our sample was high (Cronbach’s α = .76 and .77 at Time 1 and Time 2, respectively). In the current study, an anxiety composite score was created at Time 1 by calculating a mean of the five remaining SCAS subscale scores, namely generalized anxiety (6 items), panic/agoraphobia (9 items), separation anxiety (6 items), social anxiety (6 items) and physical injury fears (5 items) (Cronbach’s α = .86).

#### Depression

Depressive symptoms were assessed using the self‐report Short Mood and Feelings Questionnaire (SMFQ; Angold et al., 1995). The measure comprises 13 items which can be summed to create a total depression score. A four‐point response format was used to enable discrimination at the lower end of the spectrum (see Eley et al., 2004), giving a total score of 0–39. The SMFQ has good internal consistency and test–retest reliability and correlates well with other measures of depression (.67 with the Children’s Depression Inventory) (Angold et al., 1995). In the current study, we included SMFQ scores at Time 1 only (Cronbach’s α = .90).

### Statistical analyses

#### Phenotypic analyses

A series of linear regression models were used to test the longitudinal phenotypic associations between anxiety sensitivity and OCS. The first model estimated the proportion of the variance in OCS at Time 2 that was predicted by anxiety sensitivity at Time 1. The second model additionally included OCS at Time 1, thus testing whether anxiety sensitivity at Time 1 predicted *change in OCS* (i.e. residual variance in OCS at Time 2 after accounting for OCS at Time 1). The third regression model determined the extent to which anxiety sensitivity predicted change in OCS after controlling for anxiety and depression at Time 1, thereby testing the specificity of the association. Lastly, the bidirectionality of the longitudinal relationship between anxiety sensitivity and OCS was examined using a cross‐lagged model. The cross‐lagged model estimated the strength of the longitudinal association from anxiety sensitivity at Time 1 to OCS at Time 2, and from OCS at Time 1 to anxiety sensitivity at Time 2, while controlling for the concurrent link between anxiety sensitivity and OCS at Time 1.

Linear regressions were conducted in STATA version 14.1, using the robust cluster option to account for the nonindependence of twins/siblings. The cross‐lagged model was conducted using the OpenMx package in R (Boker et al., 2011). All variables showed evidence of positive skew and so were log‐transformed prior to analyses (see Table S1 for skewness of raw and transformed variables). Analyses controlled for age and sex.

#### Genetic analyses

The aetiology of associations between anxiety sensitivity and OCS was explored using multivariate genetic models. The twin design compares the degree of phenotypic similarity between MZ twins, who share 100% of their genes, with DZ twins and FS, who shared 50% of their segregating genes on average (Rijsdijk & Sham, 2002). Within‐pair correlations for MZ twins are compared with those for DZ twins and FS in order to estimate the effects of additive genetic factors (A); shared environment (C), defined as nongenetic factors that increase phenotypic similarity between siblings; and nonshared environment (E), defined as nongenetic factors that give rise to phenotypic differences between siblings. Greater MZ compared to DZ/FS phenotypic similarity is attributed to genetic effects. Within‐pair similarity that is not accounted for by genetic factors is attributed to shared environmental effects. Nonshared environmental effects, which are estimated from the within‐pair differences between MZ twins, also include measurement error. The sample principles can be extended to multivariate twin models, to estimate the aetiology of associations between variables. Multivariate models are based on cross‐twin cross‐trait correlations (e.g. the correlation between twin 1’s score on the first trait and twin 2’s score on the second trait). Higher cross‐twin cross‐trait correlations for MZ compared to DZ twins indicate genetic influence on covariance of the two traits.

An ACE cross‐lagged model (Malanchini et al., 2017) was used to decompose longitudinal associations between Time 1 and Time 2 variables into A, C and E paths, while controlling for the A, C and E correlations between variables at Time 1. By identifying these A, C and E paths, the model enabled us to calculate the proportion of the phenotypic longitudinal associations between anxiety sensitivity and OCS that were accounted for by genetic, shared and nonshared environmental influences (for details, see Malanchini et al., 2017). Genetic modelling was conducted within R using OpenMx (Boker et al., 2011). All variables were regressed on age and sex prior to analysis as is standard in twin modelling to avoid artificial inflation of MZ versus DZ/FS correlations (McGue & Bouchard, 1984). Models were fitted using raw data full information maximum likelihood. The fit statistic provided by OpenMx for raw data modelling is minus twice the log likelihood (−2LL) of the observations, which provides a relative measure of fit. The difference in −2LL (which is chi‐square distributed) and difference in degree of freedom between models is used to examine the overall fit of a model. Significance of parameters is established by 95% maximum likelihood confidence intervals.

## Results

Mean scores for study measures are shown in Table 1. Of note, mean scores for anxiety sensitivity and OCS decreased from Time 1 to Time 2 (anxiety sensitivity: *t*(1,564) = 25.13, *p *< .001, Cohen’s *d* = .58; OCS: *t*(1,569) = 11.27, *p *< .001, Cohen’s *d* = .26). Within‐trait and across‐trait twin correlations are shown in Table S2.

**Table 1:** Mean scores on study measures at Time 1 and Time 2 (standard deviations in parentheses)

	Time 1	Time 2
SCAS		
OCS subscale score	3.82 (3.18)	3.06 (2.95)
Anxiety composite score	4.00 (2.83)	N/A
CASI score	28.73 (5.54)	25.67 (5.73)
SMFQ score	8.07 (6.65)	N/A

Summary statistics are presented on untransformed and unregressed variables for comparison with other published samples.

CASI, Children’s Anxiety Sensitivity Index; OCS, obsessive–compulsive symptoms; SCAS, Spence Children’s Anxiety Scale; and SMFQ, Short Mood and Feelings Questionnaire.

### Does anxiety sensitivity prospectively predict OCS?

Longitudinal associations between anxiety sensitivity and OCS were initially examined using linear regression models (see Table 2). Univariate regression showed that anxiety sensitivity at Time 1 positively predicted OCS symptoms at Time 2 (β = .33, 95% CI = .28–.39, *p *< .001). This association remained significant after controlling for OCS at Time 1 (β = .12, 95% CI = .06–.17, *p *< .001) and after for controlling for OCS, anxiety and depression at Time 1 (β = .07, 95% CI = .01–.13, *p *< .05), thereby demonstrating that anxiety sensitivity predicts change in OCS independent of anxiety and depression.

**Table 2:** Results of linear regression models predicting OCS severity at Time 2 from anxiety sensitivity at Time 1

	OCS severity at Time 2	
	β	*R* ^2^
Univariate model		
AS at Time 1	.33 (.28–.39)***	.10
Multivariate model I		
OCS at Time 1	.41 (.36–.47)***	.22
AS at Time 1	.12 (.06–.17)***	
Multivariate model II		
OCS at Time 1	.37 (.31–.43)***	
Anxiety at Time 1	.07 (−.01–.15)	.23
Depression at Time 1	.05 (−.01–.12)	
AS at Time 1	.07 (.01–.13)*	

95% confidence intervals in parentheses. Analyses were conducted using robust clustering for twin pairs; all analyses adjusted for age and sex.

β, standardized coefficient; AS, anxiety sensitivity; and OCS, obsessive–compulsive symptoms.

*p < .05; ***p < .001

### Is there a reciprocal relationship between anxiety sensitivity and OCS?

**Figure 1:**
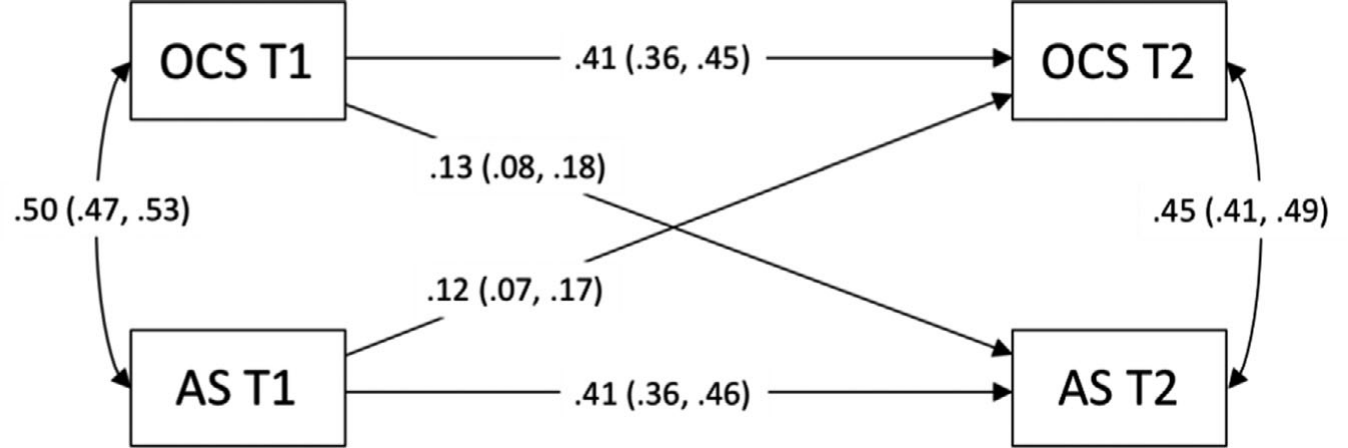
Standardized path estimates of the phenotypic cross‐lagged model showing the associations between variables over time *Note:* OCS, obsessive–compulsive symptoms; AS, anxiety sensitivity; T1, Time 1; and T2, Time 2. Values on single‐headed arrows from Time 1 to Time 2 variables are standardized partial regression coefficients. These coefficients can be squared to calculate the proportion of variance explained. Values on double‐headed arrows between variables within the same time point are correlation coefficients; the correlation at Time 2 is a residual, indexing the relationship between OCS and anxiety sensitivity at Time 2 that is not explained by their association at Time 1. 95% confidence intervals in parentheses

Bidirectionality of the longitudinal relationship between anxiety sensitivity and OCS was examined using a phenotypic cross‐lagged model (see Figure 1). The path estimates shown in Figure 1 between Time 1 and Time 2 variables demonstrate their association, controlling for the pre‐existing relationship between OCS and anxiety sensitivity at Time 1. Our results show substantial stability of both OCS and anxiety sensitivity over time (OCS: .41, 95% CI = .36–.45; anxiety sensitivity: .41, 95% CI = .36–.45). Importantly, the path from anxiety sensitivity at Time 1 to OCS at Time 2 was significant (.12, 95% CI = .07–.17). Furthermore, the path from OCS at Time 1 to anxiety sensitivity at Time 2 was also significant and of similar magnitude (.13, 95% CI = .08–.18).

### To what extent do genetic and environmental factors account for the longitudinal links between anxiety sensitivity and OCS?

Cross‐sectional genetic and environmental links between anxiety sensitivity and OCS were initially examined using correlated‐factor models, which demonstrated high genetic overlap and moderate nonshared environmental overlap between these variables at both Time 1 and Time 2 (Figure S1). Next, an ACE cross‐lagged model (Malanchini et al., 2017) was used to decompose phenotypic longitudinal associations between anxiety sensitivity and OCS into genetic (A), shared environmental (C) and nonshared environmental (E) influences. The model provided a good fit to the data (see Table S3). Although estimates of C were nonsignificant, given the modest sample size we may have been underpowered to estimate this parameter for anxiety sensitivity at Time 1 and Time 2 and therefore did not drop it from the model as this could have artificially inflated our estimates of A.

**Figure 2:**
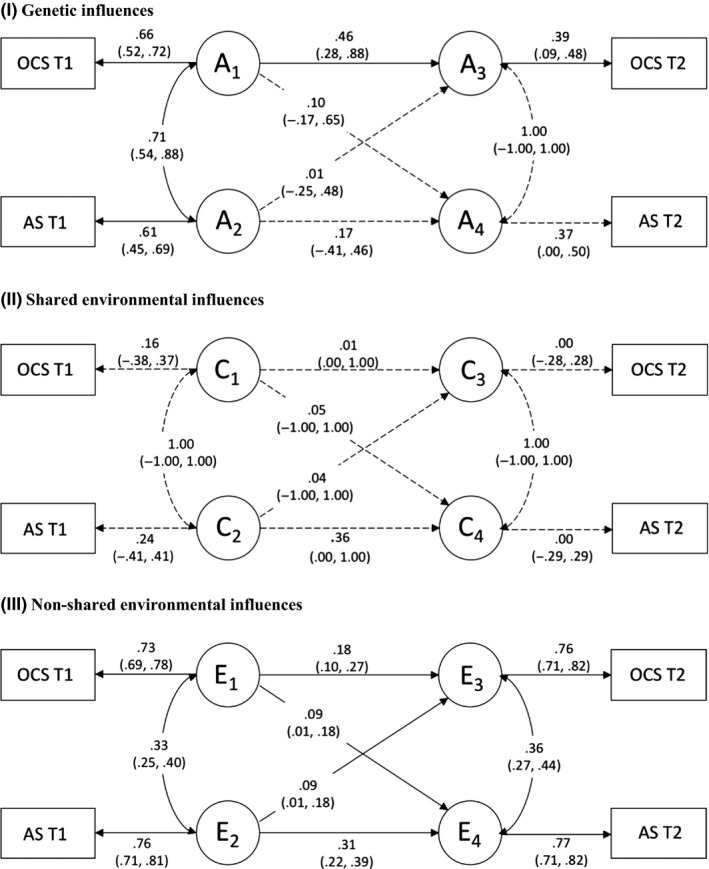
ACE cross‐lagged model showing genetic and environmental influences on anxiety sensitivity and OCS over time *Note:* OCS, obsessive–compulsive symptoms; AS, anxiety sensitivity; T1, Time 1; T2, Time 2; A, additive genetic effects; C, shared environmental effects; and E, nonshared environmental effects. Values on single‐headed arrows are standardized path estimates; values on double‐headed arrows are correlation coefficients. Genetic and environmental correlations at Time 2 are residuals, representing the relationship between anxiety sensitivity and OCS at Time 2 that is independent of their association at Time 1. 95% confidence intervals in parentheses. Solid lines represent significant paths; dashed lines represent nonsignificant paths

Results from the ACE cross‐lagged model are shown in Figure 2. The figure shows a large genetic correlation been anxiety sensitivity and OCS at Time 1 (.71, 95% CIs .88–.54), suggesting substantial overlap in the genes that influence these phenotypes. After controlling for this genetic correlation, the genetic influences on anxiety sensitivity at Time 1 did not significantly predict OCS at Time 2 (.01, 95% CIs −.25 to .48). Similarly, the genetic influences on OCS at Time 1 had a small and nonsignificant effect on anxiety sensitivity at Time 2, after controlling for the genetic correlation with anxiety sensitivity at Time 1 (.10, 95% CIs −.17 to .65). A somewhat different pattern of results was observed for nonshared environmental influences. The nonshared environmental correlation between anxiety sensitivity and OCS at Time 1 was moderate (.33, 95% CIs .25–.40), indicating modest overlap in the nonshared environmental risk factors for these phenotypes. After controlling for this overlap, the nonshared environmental influences on OCS at Time 1 had a small but significant effect on anxiety sensitivity at Time 2 (.09, 95% CIs .01–.18). Likewise, the nonshared environmental influences on anxiety sensitivity at Time 1 had a small but significant effect on OCS at Time 2 (.09, 95% CIs .01–.18).

The path estimates shown in Figure 2 were used to calculate the proportion of stable and cross‐lagged covariance that was accounted for by A, C, and E (see Table 3). The cross‐lagged association between anxiety sensitivity at Time 1 and OCS at Time 2 was almost entirely attributable to nonshared environmental factors (95%), with genetic influences accounting for a negligible proportion of the covariance (5%). The cross‐lagged association between OCS at Time 1 and anxiety sensitivity at Time 2 was also largely accounted for by nonshared environmental influences (70%), with genetic factors explaining a moderate but nonsignificant proportion of the relationship (30%).

**Table 3:** Path estimates for the stability and cross‐lagged paths and the proportion accounted for by genetic (A), shared environmental (C) and nonshared environmental (E) influences

	Phenotypic path estimate	Percentage of covariance explained		
		A	C	E
Stability paths				
OCS T1 → OCS T2	.41 (.36–.45)	.54 (.16–1.00)	.00 (.00–.54)	.46 (.27–1.00)
AS T1 → AS T2	.41 (.36–.46)	.18 (.00–1.00)	.00 (.00–.57)	.82 (.36–1.00)
Cross‐lagged paths				
OCS T1 → AS T2	.13 (.08–.18)	.30 (.00–1.00)	.00 (.00–.79)	.70 (.09–1.00)
AS T1 → OCS T2	.12 (.07–.17)	.05 (.00–1.00)	.00 (.00–1.00)	.95 (.43–1.00)

A = additive genetic effects; C = shared environmental effects; and E = nonshared environmental effects. 95% confidence intervals in parentheses.

AS, anxiety sensitivity; OCS, obsessive–compulsive symptoms; T1, Time 1; and T2, Time 2.

## Discussion

The current study represents the largest investigation of anxiety sensitivity and OCS to date, and the first using genetically informative data. In relation to our first aim, we found that anxiety sensitivity during mid‐adolescence positively predicted changes in OCS over a two‐year period. The prospective relationship between anxiety sensitivity and OCS was partly accounted for by coexisting anxiety and depression, but nevertheless remained significant even after controlling for these symptoms. In this stringent test, the magnitude of the association between earlier anxiety sensitivity and later OCS was small, but this is not surprising given that our analysis accounted for the stable, more reliable variance. Furthermore, a range of other factors could have attenuated the effect (e.g. wide age range of participants at each time point, measurement error). Nevertheless, the only previous study that has examined the prospective relationship between anxiety sensitivity and changes in OCS found a similar effect size (Schmidt et al., 2010). Although statistically significant, it is important to note that anxiety sensitivity *alone* may not be a clinically meaningful predictor of OCS, given the modest strength of association. However, anxiety sensitivity could potentially be combined with other variables to create a risk index for OCS, an approach which has been taken in several related fields (Hudson et al., 2013; Meehan et al., 2019).

With respect to our second aim, we also found evidence for a reciprocal relationship between anxiety sensitivity and OCS over time. Thus, while anxiety sensitivity may be a risk factor for OCS, the reverse association appears to be true with OCS conferring risk for heightened anxiety sensitivity. This novel finding is similar to the results of a previous study, which showed a bidirectional relationship between anxiety sensitivity and anxiety symptoms (Zavos et al., 2012), and raises the possibility that individuals could become trapped in a vicious cycle of escalating anxiety sensitivity and OCS.

In relation to our third aim, the current study demonstrated that the reciprocal links between anxiety sensitivity and OCS over time are largely accounted for by nonshared environmental factors. This does not mean that genetic factors do not influence the relationship between anxiety sensitivity and OCS, and in fact we demonstrated a high level of genetic overlap between anxiety sensitivity and OCS. However, our findings show that the extent to which anxiety sensitivity predicts *change* in OCS over time is almost entirely attributable to nonshared environmental influences. Thus, individuals with heightened anxiety sensitivity may respond differently to life experiences, rendering them more vulnerable to OCS. For example, heightened anxiety sensitivity could elicit changes in parenting (e.g. overprotective parenting), modify anxiety‐relevant learning and/or amplify anxious responses to stressful life events, leading to increased OCS risk. Similarly, the current study shows that the extent to which OCS predicts *change* in anxiety sensitivity is largely accounted for by the nonshared environment. Thus, it is possible that experiencing frequent anxious arousal in the context of heightened OCS may lead to an aversion to the physiological sensations of anxiety. Indeed, sustained exposure to stressful environments (e.g. military training) has been shown to predict increases in anxiety sensitivity (Schmidt et al., 2000).

There are several theoretical and clinical implications of the current findings. Our results are consistent with the hypothesis that anxiety sensitivity is one of a myriad of risk factors for OCS. Future studies should seek to understand the mechanisms underpinning the prospective association between anxiety and OCS, and investigate how it relates to other psychological constructs which may be risk factors for OCS, such as distress intolerance (Robinson & Freeston, 2014). Our findings suggest that heightened anxiety sensitivity could be a marker for identifying adolescents at risk of developing OCS, in conjunction with other known risk factors (e.g. family history of OCD). Moreover, anxiety sensitivity could be one potential target for OCD prevention. Encouragingly, anxiety sensitivity can effectively be reduced with a single‐session psychological intervention (Schmidt, Capron, Raines, & Allan, 2014), and effective reduction of anxiety sensitivity has been shown to ameliorate OCS in nonclinical samples (Timpano, Raines, Shaw, Keough, & Schmidt, 2016). The efficacy of such interventions in the prevention of OCD warrants further investigation. Lastly, our findings also highlight the value of early intervention for OCS. Not only are OCS impairing in their own right (Fullana et al., 2009), but effective treatment could lower risk for developing elevated anxiety sensitivity, which could in turn reduce risk for a wide range of other anxiety subtypes (Waszczuk et al., 2013).

This study has several strengths, including the prospective, genetically informative design and the inclusion of measures of anxiety and depression to control for comorbid psychopathology. However, findings should be interpreted in the context of a number of limitations. When we repeated the current analyses with a later wave of data, the pattern of results was somewhat different (see Tables S4, S5 and Figures S2, S3). Anxiety sensitivity at Time 2 predicted change in OCS by Time 3 (3 years 4 months later on average), but this association did not remain when controlling for anxiety and depressive symptoms. Instead, we found a significant cross‐lagged association between anxiety symptoms and OCS at Time 2 and Time 3. Discrepant findings could be accounted for by a range of factors including differences in ages and time intervals between assessment points, as well as substantial participant attrition/missing data at Time 3 (23%). Nevertheless, anxiety sensitivity and anxiety symptoms are closely related (*r *= .68 and *r *= .69 at Time 1 and Time 2, respectively), and overall, our findings support the notion that the broader anxiety phenotype may be a risk factor for OCS. Future research should explore whether there are differential effects of anxiety sensitivity versus anxiety symptoms on OCS at different stages of development.

In addition, several other limitations of the current study should be considered. First, only adolescent self‐report measures were included. Reliance on a single informant may have inflated associations, and it will be important for future research to include parent‐report and/or clinician‐administered measures. Second, the current study focused on OCS in nonclinical participants. It cannot be assumed that the findings would generalize to diagnosable OCD, although the use of nonclinical samples to study OCD is well supported (Abramowitz et al., 2014). Third, the wide age range of participants at each time point means that the developmental significance of our findings should be interpreted cautiously. Fourth, our genetic cross‐lagged model had limited statistical power and therefore findings should be viewed as preliminary. Fifth, the current study has a number of limitations that are inherent to twin design studies, including the assumption of equal environments across zygosities and the generalizability of twins to the general population (Rijsdijk & Sham, 2002).

In summary, our findings indicate a prospective association between anxiety sensitivity and OCS during adolescence. Furthermore, our results suggest that anxiety sensitivity and OCS mutually influence each other over time. These reciprocal links do not appear to be accounted for by a shared genetic vulnerability, but instead are more likely accounted for by nonshared environmental factors. If replicated, our findings raise the possibility that interventions aimed at ameliorating anxiety sensitivity could reduce risk for OCS, but also that early intervention for OCS could lower risk for anxiety sensitivity and subsequent anxiety psychopathology.

## Supporting information


**Table S1** **.** Skewness of variables before and after log transformation.
**Table S2** **.** Twin correlations within–trait, across‐trait and across‐time (cross‐lag model).
**Table S3** **.** Fit comparisons for ACE cross‐lagged model.
**Table S4** **.** Mean scores on study measures at Time 3 (standard deviations in parentheses).
**Table S5** **.** Results of linear regression models predicting OCS severity at Time 3 from anxiety sensitivity at Time 2.
**Figure S1** **.** Multivariate correlated factors models showing genetic and environmental influences on anxiety sensitivity, obsessive‐compulsive, anxiety and depressive symptoms at Time 1/Time 2.
**Figure S2** **.** Standardized path estimates of the phenotypic cross‐lagged model showing the associations between variables over time.
**Figure S3** **.** AE cross‐lagged model showing genetic and environmental influences on anxiety sensitivity and OCS over time.
