# Supplementary material for: Reciprocal links between anxiety sensitivity and obsessive–compulsive symptoms in youth: a longitudinal twin study
Source: J Child Psychol Psychiatry. 2020 Jan 16;61(9):979–87. doi: 10.1111/jcpp.13183 (PMC7497024; doi:10.1111/jcpp.13183)
Supplement: Supplementary file 1 — Table S1 . Skewness of variables before and after log transformation. Table S2 . Twin correlations within–trait, across‐trait and across‐time (cross‐lag model). Table S3 . Fit comparisons for ACE cross‐lagged model. Table S4 . Mean scores on study measures at Time 3 (standard deviations in parentheses). Table S5 . Results of linear regression models predicting OCS severity at Time 3 from anxiety sensitivity at Time 2. Figure S1 . Multivariate correlated factors models showing genetic and environmental influences on anxiety sensitivity, obsessive‐compulsive, anxiety and depressive symptoms at Time 1/Time 2. Figure S2 . Standardized path estimates of the phenotypic cross‐lagged model showing the associations between variables over time. Figure S3 . AE cross‐lagged model showing genetic and environmental influences on anxiety sensitivity and OCS over time. [file JCPP-61-979-s001.docx]

**Table S1: Skewness of variables before and after log transformation.**

| **Variable** | **Skewness of untransformed variable** | **Skewness of log transformed variable** |
| --- | --- | --- |
| OCS at Time 1 | 1.19 | .34 |
| OCS at Time 2 | 1.55 | .12 |
| Anxiety sensitivity at Time 1 | .60 | .10 |
| Anxiety sensitivity at Time 2 | 1.16 | .64 |
| Anxiety composite at Time 1 | 1.00 | .34 |
| Depression at Time 1 | 1.35 | .45 |

*Note:* OCS = obsessive-compulsive symptoms

**Table S2: Twin correlations within–trait, across-trait and across-time (cross-lag model).**

|  |  |  | Twin 2 | | | |
| --- | --- | --- | --- | --- | --- | --- |
|  |  |  | OCS T1 | AS T1 | OCS T2 | AS T2 |
| Monozygotic | |  |  |  |  |  |
|  | Twin 1 | OCS T1 | .47 | .32 | .31 | .18 |
|  |  | AS T1 | - | .45 | .21 | .19 |
|  |  | OCS T2 | - | - | .39 | .29 |
|  |  | AS T2 | - | - | - | .29 |
|  |  |  |  |  |  |  |
| Dizygotic | |  |  |  |  |  |
|  | Twin 1 | OCS T1 | .24 | .18 | .16 | .13 |
|  |  | AS T1 | - | .21 | .12 | .17 |
|  |  | OCS T2 | - | - | .16 | .13 |
|  |  | AS T2 | - | - | - | .19 |

*Note:* OCS = obsessive-compulsive symptoms; AS = anxiety sensitivity; T1 = Time 1; T2 = Time 2

**Figure S1: Multivariate correlated factors models showing genetic and environmental influences on anxiety sensitivity, obsessive-compulsive, anxiety and depressive symptoms at Time 1/Time 2.**

Genetic influences

Shared environmental influences

Non-shared environmental influences

Note: AS = anxiety sensitivity; OCS = obsessive-compulsive symptoms; Anx = anxiety symptoms; Dep = depressive symptoms; A = additive genetic effects; C = shared environmental effects; E = non-shared environmental effects; values on single-headed arrows are standardized path estimates; values on double-headed arrows are correlation coefficients; 95% confidence intervals in parentheses.

**Table S3: Fit comparisons for ACE cross-lagged model.**

| Base model | Comparison model | -2LL | df | diff LL | diff df | *p* | AIC |
| --- | --- | --- | --- | --- | --- | --- | --- |
| Saturated model | N/A | 24670.43 | 8007 | N/A | N/A | N/A | 8656.43 |
| Saturated model | ACE model | 24741.39 | 8061 | 70.96 | 54 | .06 | 8619.39 |

*Note:* -2LL = minus twice the log likelihood; AIC = Akaike’s information criterion; A = additive genetic influence; C= shared environmental influences; E = non-shared environmental influences.

**Table S4: Mean scores on study measures at Time 3 (standard deviations in parentheses).**

|  | Time 3  (*n* = 1182 – 1214) |
| --- | --- |
| RCADS |  |
| OCS subscale score | 3.13 (3.10) |
| Anxiety composite score | 5.49 (3.20) |
| ASI score | 15.5 (9.41) |

*Note:* Revised Children’s Anxiety and Depression Scale; OCS = obsessive-compulsive symptoms; ASI = Anxiety Sensitivity Index. The ASI comprises 16 items. The RCADS includes 6 OCS items and 30 anxiety items.

Summary statistics are presented on untransformed and unregressed variables for comparison with other published samples.

**Table S5:** **Results of linear regression models predicting OCS severity at Time 3 from anxiety sensitivity at Time 2.**

|  | OCS severity at Time 2 | |
| --- | --- | --- |
|  | β | R^2^ |
| **Univariate model** |  |  |
| AS at Time 1 | 36 (.30 - .41) *** | .13 |
| **Multivariate model I** |  |  |
| OCS at Time 1 | .48 (.42 - .54) *** | .29 |
| AS at Time 1 | .08 (.02 - .14) ** |  |
| **Multivariate model II** | |  |
| OCS at Time 1 | 43 (.36 - .49) *** | .30 |
| Anxiety at Time 1 | .13 (.05 - .21) ** |  |
| Depression at Time 1 | .06 (-.00 - .13) ^ns^ |  |
| AS at Time 1 | -.01 (-.08 - .06) ^ns^ |  |

*Note:* AS = anxiety sensitivity; OCS = obsessive-compulsive symptoms; β = standardized coefficient; 95% confidence intervals in parentheses. Analyses were conducted using robust clustering for twin pairs; all analyses adjusted for age and gender.

* *p* < .05	** *p* < .01	*** *p* < .001

**Figure S2:** **Standardized path estimates of the phenotypic cross-lagged model showing the associations between variables over time.**

*Note:* OCS = obsessive-compulsive symptoms; AS = anxiety sensitivity; ANX = anxiety symptoms; T2 = time 2; T3 = time 3. Values on single-headed arrows from Time 2 to Time 3 variables are standardized partial regression coefficients. These coefficients can be squared to calculate the proportion of variance explained. Values on double-headed arrows between variables within the same timepoint are correlation coefficients; the correlation at Time 3 is a residual, indexing the relationship between OCS and anxiety sensitivity at Time 3 that is not explained by their association at Time 2. 95% confidence intervals in parentheses.

**Figure S3:** **AE cross-lagged model showing genetic and environmental influences on anxiety sensitivity and OCS over time.**

**Genetic influences**

**Non-shared environmental influences**

*Note:* OCS = obsessive-compulsive symptoms; AS = anxiety sensitivity; ANX = anxiety symptoms; T2 = time 2; T3= time 3; A= additive genetic effects; E = non-shared environmental effects. Values on single-headed arrows are standardized path estimates; values on double-headed arrows are correlation coefficients; 95% confidence intervals in parentheses. Solid lines represent significant paths; dashed lines represent non-significant paths.
